# Exposure to Dichlorodiphenyldichloroethylene (DDE) and Metallothionein Levels in Rats Fed with Normocaloric or High-Fat Diet: A Review

**DOI:** 10.3390/ijms21051903

**Published:** 2020-03-10

**Authors:** Vincenzo Migliaccio, Lillà Lionetti, Rosalba Putti, Rosaria Scudiero

**Affiliations:** 1 Department of Chemistry and Biology “Adolfo Zambelli”, University of Salerno, 84084 Fisciano (Sa), Italy; vmigliaccio@unisa.it (V.M.); llionetti@unisa.it (L.L.); 2 Department of Biology, University Federico II, 80126 Napoli, Italy; rosalba.putti@unina.it

**Keywords:** dichlorodiphenyltrichloroethane (DDT), dichlorodiphenyldichloroethylene (DDE), metallothionein, saturated fatty acids, Wistar rat tissues, zinc availability

## Abstract

The growing number of studies on metallothioneins (MTs), cysteine-rich metal-binding proteins, have been disclosing new functions of these proteins. Thanks to their inducibility, they were considered to play a pivotal role in regulating trace metals homeostasis and in detoxification from heavy metals; nowadays, it is known that they are involved in various physiological and pathological processes, such as regulation of apoptosis, elimination of free radicals, and protection of nucleic acids against toxic insults. MT induction has been demonstrated following stress factors other than heavy metals, such as endocrine-disrupting chemicals, insecticides, and herbicides. However, retrieved data are often controversial: in some cases, xenobiotics elicit MT expression and synthesis; under different conditions, they lead to a decrease in cellular MT content. This review describes the MT response to dichlorodiphenyltrichloroethane (DDT) contamination in mammalian tissues. In particular, attention focuses on changes in MT expression, synthesis, and localization in rat liver, kidneys, and testes following oral administration of dichlorodiphenyldichloroethylene (DDE), the main metabolite of DDT, under normal dietary conditions or in combination with a high fat diet potentially able to increase the cellular uptake of this lipophilic pesticide. The potential connection between MT expression and synthesis, lipophilic substances and trace metals availability is also discussed.

## 1. Multifunctional Roles of Metallothioneins

Since their discovery in the equine renal cortex in 1956 [1], metallothioneins (MTs) are known especially for their detoxifying properties against toxic heavy metals [2]. In fact, they belong to a multigene family of proteins able to form metal-thiolate bonds thanks to the high number of cysteines present in their amino acid chain (about 30% of total amino acids) [3]. Cytosolic proteins are ubiquitously expressed in almost all animal cell types [4,5]; under physiological conditions, they bind to essential metals, such as zinc (Zn) and copper (Cu), thus forming a reserve of these micronutrients immediately available in the cells; they also exhibit high affinity for toxic heavy metals without any biological function, such as mercury (Hg), lead (Pb), and cadmium (Cd) [6]. The fundamental hallmark for carrying out this detoxifying function is the ability of MTs to be induced by multiple heavy metal species at the transcriptional level thanks to the presence of multiple copies of metal-responsive elements (MRE) in the upstream region of MT-encoding genes [7,8].

The growing number of studies of MTs has disclosed many other roles of these proteins, especially in mammals, including humans. In mammals, the MT family consists of 4 subgroups, namely, MT1-MT4. MT1 and MT2 are further divided into multiple isoforms, often different from each other only in untranslated gene regions and found in almost all tissues [9]; MT3 and MT4 are each a single isoform typical for neurons and epithelial cells, respectively [10,11]. Although MT1 and MT2 isoforms are considered almost equivalent in their functions being both inducible by the same stimuli (metals, hormones, oxidative stress, etc.), evidence exists on a different metal selectivity between the two isoforms [12,13,14]. This suggests a changeable biological function for MT1 and MT2, where the metal bound to the protein could play an important physiological role. For example, in a mouse brain, a greater protective capacity of the MT2 isoform compared to the MT1 isoform has been demonstrated against the demyelination process induced by encephalomyelitis, probably due to the stronger Zn–thionein character of the MT2 isoform [15]. However, it has been demonstrated that all MT isoforms are involved in many cellular responses to pathological conditions, such as cancer, inflammation, oxidative stress [16,17,18,19,20,21]. Hence, MTs are now assumed to be multifunctional proteins with additional unidentified physiological roles. The molecular outline of MT gene expression showed the ability of the reactive oxygen species (ROS) to induce production of the MTs, which in turn act as scavengers, reducing the toxic effect of free radicals, especially at the DNA level [22,23].

For their inducibility and their involvement in cellular responses to stress factors, nowadays MTs are considered excellent biomarkers for assessing environmental pollution not only by heavy metals [24,25,26,27], but also by many other substances of known or uncertain cellular toxicity, such as insecticides and herbicides [28,29,30,31,32,33]. For example, the exposure to the synthetic pyrethroid deltamethrin increased the MT mRNA expression levels in rainbow trout muscles [30]. The exposure to the herbicide isoproturon induces the MT expression in the aquatic oligochaeta *Tubifex tubifex* [31]. In vertebrates, it has been observed that MT expression increases in tissues of mice exposed to the herbicide paraquat [32], similarly to that observed in the lizard liver after treatment with the herbicide glyphosate [33]. However, a comprehensive survey on the involvement of MTs in cellular stress injury induced by pesticides is yet to appear. The current review summarizes the recent knowledge of MT response to dichlorodiphenyltrichloroethane (DDT) contamination in mammalian tissues. The literature search up to December 2019 was performed on the electronic databases PubMed, Scopus, and Web of Science with the following search keywords: “metallothionein” AND “Dichloro–Diphenyl–Trichloroethane”, “metallothionein” AND “pesticides”.

## 2. Metallothioneins and DDT Metabolites

DDT [1,1′-(2,2,2-trichloroethane-1,1-diyl)bis(4-chlorobenzene)], also known as dichlorodiphenyltrichloroethane, is a synthetic organochlorine low-cost pesticide widely used in the past against malaria [34,35]. Though banned in rich industrialized countries since 1970s, DDT contamination of soils is particularly widespread due to its persistence [36]. In addition, it is still used in developing countries, where malaria and other diseases transmitted by insects are endemic [37]; the grasshopper effect contributes to the DDT dispersion on all continents [38].

Once in the soil, it is partly metabolized to dichlorodiphenyldichloroethylene (DDE) and dichlorodiphenylethane (DDD) [39]; the three different formulations can enter the food chain reaching the top and causing cellular diseases in many animal species [40]. Being lipophilic, DDT and its derivatives accumulate in fatty tissues [41]; as a result, humans inhabiting richer industrialized countries that have banned DDT are evenly exposed, as they generally follow high-calorie, meat-rich diets [42].

It has been demonstrated that the presence of DDT and its metabolites in animal tissues causes oxidative stress and mitochondrial damage [43,44,45,46,47,48], whereas few data, often controversial, are available on the effects of these substances on MT expression and synthesis. A study performed by Ben Miled and coworkers [49] showed an increase in MT content in hepatocytes of rats receiving a single intraperitoneal injection of 100 mg DDT/kg body weight (bw). In kidneys and testes of rats receiving the same DDT dose for ten days, MT content significantly decreased [45,46].

The present review focuses on the results obtained from the studies that aimed to clarify a possible involvement of MTs in cellular responses elicited by DDE, the major DDT metabolite, when administered orally. DDE-induced changes of MT expression and synthesis were assessed in three rat organs: in the liver and kidneys, the main organs involved in the detoxification and excretion of toxic substances known to be rich in MTs [50], and in testes, a well-studied target of the toxic action of DDT and DDE [45,51,52]. Knowing the effect of intraperitoneally administered DDT on MT synthesis in rat kidneys and testes [45,46], the effect on cellular localization of MTs, gene expression, and protein synthesis was determined following a 4-week-long oral administration of DDE (10 mg/kg bw) added to a normocaloric diet (10.6% fat J/J, 15.47 KJ/g) or to a high-calorie diet (45% fat J/J, 19.88 KJ/g) rich in saturated fatty acids and able to promote DDE accumulation in cells.

The oral administration of DDE at this dose for 4 or 6 weeks does not affect physical development, sexual maturation, and serum metabolic parameters of male rats [53].

## 3. Effect of DDE on Expression, Synthesis, and Localization of MTs in Rat Tissues

### 3.1. Liver

The constitutive amount of MT mRNAs in tissues of male Wistar rats of 2 months of age was determined by real-time PCR analysis [54,55,56]. The MT primers used allow to amplify and quantify together both MT1 and MT2 isoforms [55,56]. As expected, MT transcripts were present in all the three tissues examined, the order of abundance of transcripts being as follows: liver > testes > kidneys. In particular, the relative amounts of MT transcripts were almost comparable between liver and testes; in kidneys, the MT mRNA level was about 5 times lower. A similar scenario was also found for MT proteins, whose relative amount in the three different organs was evaluated performing the Western blot analysis [56].

Following a 4-week-long oral administration of DDE, significant changes in MT transcript content were observed in rat tissues, also depending on the diet.

In liver, the hyperlipidemic diet alone produced a dramatic decrease in MT transcripts to less than 1/3 of the amount determined in rats fed a standard diet (Ctr rats); in the latter, DDE administration was also able to elicit the downregulation of MT genes in hepatocytes (Ctr + DDE rats). An equivalent amount of MT transcripts was found in livers of the rats fed a high-fat diet (HF rats) and in the rats with a high-fat diet plus oral DDE administration (HF + DDE rats).

Western blot analysis retrieved a similar scenario for MT protein content in liver homogenate, with the highest MT level observed in the rats fed a normocaloric diet (Ctr group); the lowest MT protein content was detected in livers of the rats belonging to the HF + DDE group (Table 1).

**Figure 1:**
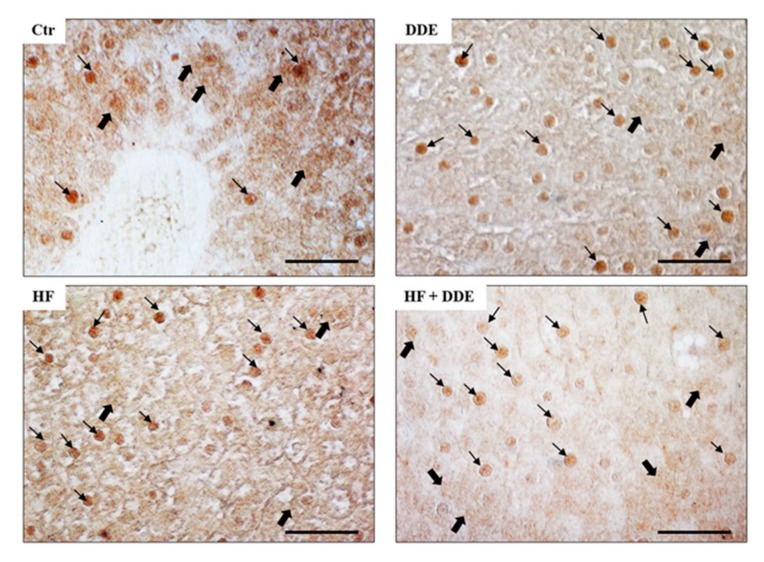
Metallothionein (MT) localization in rat liver. In control animals (Ctr), the immunocytochemical signal (brown areas) was detected in cell cytoplasm (thick arrows) and in some nuclei (thin arrows). In all the treated animals, the immunoreactive signal was detected in a larger number of nuclei, whereas the cytosolic localization of MTs was less prevalent. HF, high-fat diet. Bar = 50 µm. Adapted from Migliaccio et al., 2019 [56].

Noteworthy, the increase in saturated fatty acids intake and the DDE administration modified the intracellular localization of MT proteins in the hepatocytes, as demonstrated by immunocytochemical analysis (Figure 1). In control rats, positivity for MT was localized mostly in the cytosol and was less evident in nuclei; in HF and DDE-treated rats, the staining was low in the cytosol and high in nuclei of hepatocytes. These results were validated by Western blot analysis of cytosolic and nuclear extracts.

**Figure 2:**
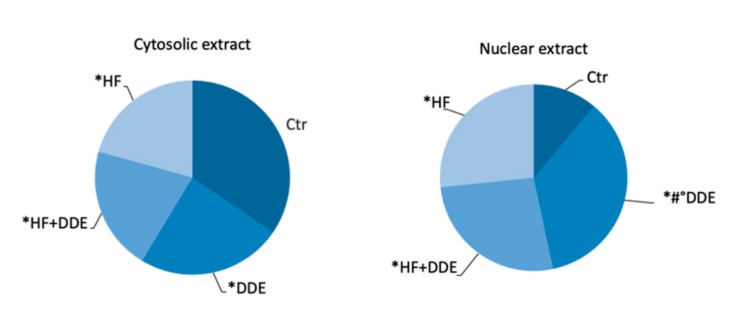
MT proteins in cytosolic and nuclear rat liver extracts. Statistical analysis was performed by using ANOVA followed by the Bonferroni post-hoc test. * *p* < 0.001 vs. Ctr, # *p* < 0.001 vs. HF, ° *p* < 0.001 vs. HF + DDE. Adapted from Migliaccio et al., 2019 [56].

The nuclear translocation of MT proteins occurred in hepatocytes of all the treated rats, with the marked increase retrieved in the rats receiving only oral administration of DDE (Figure 2).

To sum up, the data collected on rat liver demonstrated that both DDE and HF treatments downregulated MT expression and synthesis, and no synergistic or additive effects were observed between the action of pesticide and fats. This finding is quite surprising, because it is known that DDE, as well as saturated fatty acids, has a strong pro-oxidant activity that in theory was supposed to stimulate MT expression and synthesis in cells. It has been demonstrated that following the oral administration of DDE and saturated fatty acids, rat hepatocytes increase the amount and activity of antioxidant enzymes, such as superoxide dismutase (SOD), catalase (CAT), and glutathione peroxidase (GPx), thus suggesting that cells respond specifically to the oxidative stress [56]. The treatments also induce lipid peroxidation, a cellular condition previously associated with inhibition of MT synthesis in rat testes [45]. However, the nuclear translocation of MT proteins observed in the hepatocytes of the animals administered DDE or fed a high-fat diet outlines a role of MTs in the protection of DNA from hydroxyl radical attacks [57,58].

### 3.2. Kidneys

Real-time PCR analysis performed to evaluate MT1 and MT2 mRNA levels in kidneys of the rats belonging to the four experimental groups revealed a different response to the HF diet and DDE administration in this organ compared to the liver.

The high-fat diet alone slightly reduced MT gene expression, whereas DDE administration significantly increased (about nine times) the MT mRNA level as compared to the rats fed a normocaloric (Ctr) or a high-fat (HF) diet. Simultaneous exposure to DDE and a high-fat diet resulted in a slightly lower increase (about five times).

Western blotting data on total renal extracts showed the same trend for the MT mRNA level for the MT protein, i.e., a significantly higher content of MTs in DDE-treated groups. No appreciable difference in MT levels was found between DDE and HF + DDE groups (Table 1).

**Figure 3:**
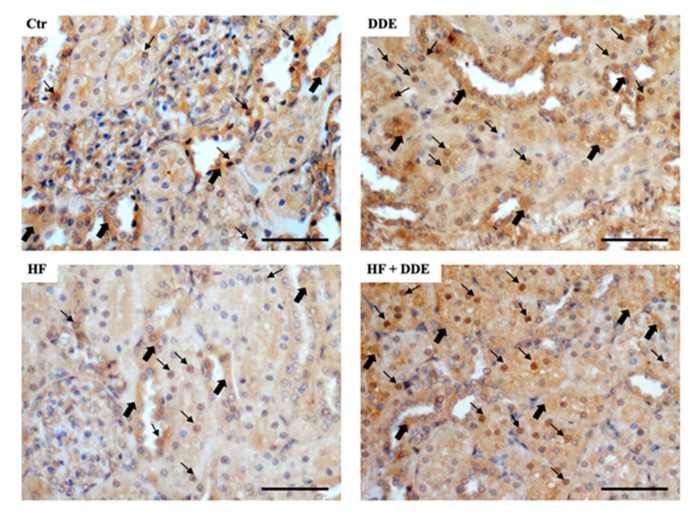
Metallothionein localization in rat kidneys. In control animals, the immunocytochemical signal was detected in the cytoplasm (thick arrows) and in rare nuclei (thin arrows). In kidney of the rats fed a high-fat diet (HF), the immunostaining was weaker, but several nuclei were clearly positive. In the DDE-treated animals, no matter the diet, a marked positivity was detected in the cytosol and in many nuclei. Nuclei were counterstained with hematoxylin. Bar = 50 µm. Adapted from Migliaccio et al., 2019 [56].

**Figure 4:**
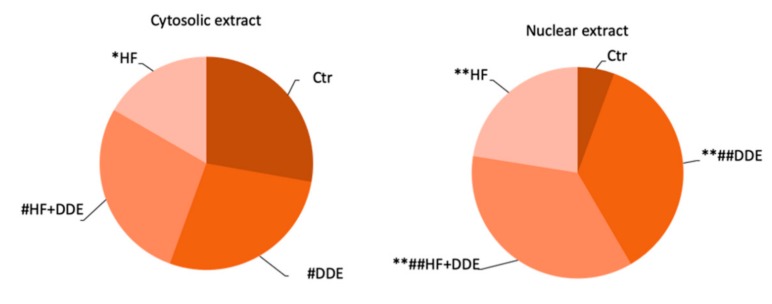
MT proteins in cytosolic and nuclear rat kidney extracts. Statistical analysis was performed by using ANOVA followed by the Bonferroni post-hoc test. * *p* < 0.01 vs. Ctr, ** *p* < 0.001 vs. Ctr, # *p* < 0.05 vs. HF, ## *p* < 0.001 vs. HF. Adapted from Migliaccio et al., 2019 [56].

As in the case of the liver, immunocytochemical (Figure 3) and Western blot analyses (Figure 4) of kidneys also demonstrated that DDE administration resulted in a change in the cellular MT distribution: the protein, primarily cytosolic under normal conditions, appeared mainly located in the nucleus.

Hence, in kidneys, both real-time PCR and Western blot analyses indicated the upregulation of MT expression and synthesis in the presence of DDE, no matter the type of the diet.

This result is opposite to that in the liver, where DDE caused MT downregulation. Since the basal level of MTs in kidneys is lower if compared with the liver, it could be hypothesized that in the renal cells, MT genes retain more responsivity to stress signals.

### 3.3. Testes

In testes, the MT expression profile showed downregulation of MT genes in all the treated groups when compared to the control group. The greatest difference was observed in testes of the rats fed a normocaloric diet receiving DDE by gavage, in which MT transcripts decreased by a half; in HF and HF + DDE groups, MT transcripts decreased by 1/3.

The Western blot analysis performed on total testicular proteins retrieved the similar trend: MT proteins decreased in testes of the rats belonging to the three treated groups if compared to the control group (DDE < HF + DDE = HF < Ctr) (Table 1).

**Figure 5:**
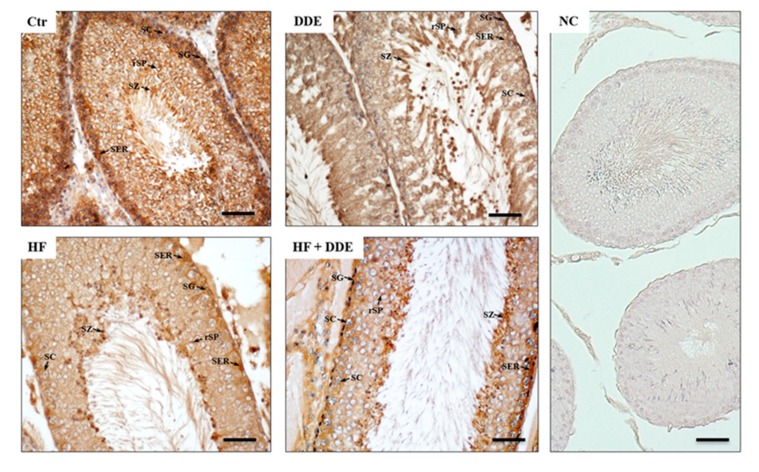
Metallothionein localization in rat testes. In control animals, the immunohistochemical signal (brown areas) was detected in the cytoplasm of all the cells forming the seminiferous epithelium: somatic Sertoli cells (SER), spermatogonia (SG), spermatocytes (SC), round spermatids (rSP), and spermatozoa heads (SZ). In all the treated animals, the immunoreactive MT signal was weak, mostly localized on Sertoli cells, spermatogonia, and spermatozoa. No signal was evident in the negative control (NC) sections incubated without the anti-MT antibody. Nuclei were counterstained with hematoxylin. Bar = 50 µm.

The immunolocalization of MT proteins in testes confirmed the results obtained with the Western blot analysis. In testes of the control rats, the immunostaining was localized throughout the surface of the seminiferous epithelium; however, the basal compartment made of spermatogonia, spermatocytes, and Sertoli cells was more intensely stained, as well as the sperm heads forming the adluminal edge (Figure 5). In testes of the treated rats, the MT localization did not change, but the immunoreactivity appeared generally reduced (Figure 5).

A rat testis contains a high level of constitutive MT transcripts, comparable to that in the liver. The fact that MT genes in testicular cells respond to DDE and/or HF administration as in hepatocytes decreasing their expression reinforced the hypothesis, according to which the MT genes are highly expressed in the liver and testes and, consequently, are less responsive to the stressful factors other than metals. Unlike in the liver and kidneys, in testes, no changes in intracellular MT distribution were observed following DDE administration and/or an HF diet, the staining being mostly cytoplasmic (Figure 5).

## 4. Concluding Remarks

Recently, histological and molecular analysis indicated that oral exposure to a moderately low dose of DDE (10 mg/kg bw) for 4 weeks was able to elicit morphological alterations, cellular oxidative stress, and lipid peroxidation in rat tissues [47,48,56,58]. Studies also evidenced the presence of similar morphological and oxidative damage caused by the fat diet alone [47,48,56,58]. In the liver, an HF diet induced steatosis of hepatocytes, and DDE treatment—tissue inflammation and cellular vacuolization. In kidneys, DDE administration caused renal tubular injuries, mainly in the proximal tubules; in testes, morphological alterations of seminiferous tubules with a consequent impairment of spermatogenesis were observed. In the three different organs, changes were recorded in the oxidative status, where an increase in lipid peroxides was observed following both the HF diet and DDE administration. It has also been demonstrated that cells activated the endogenous antioxidant system to protect themselves from the oxidative damage generated in HF- and DDE-treated animals. In particular, increased synthesis and enzymatic activity of SOD1/2 and total GPx antioxidant enzymes and the induction of the mitochondrial uncoupling protein 2 (UCP2) were observed in the hepatocytes the to limit the mitochondrial damage produced by ROS [47,56]. On the contrary, testicular cells showed antioxidant system impairment in the HF- and DDE-treated animals associated with an increase in lipid peroxidation and the apoptotic rate. However, testicular cells try to counterbalance cellular apoptosis by increasing the cellular proliferation rate [58,59]. This mechanism could be important to maintain a functional pool of seminiferous tubules that undergo physiological differentiation and maturation.

With regard to MTs, the results deserve special attention. Although it is widely assumed that the presence of both MT1 and MT2 isoforms in animal cells is associated with different biological functions, such as essential metals metabolism, heavy metals detoxification, and ROS scavenging [6], the results summarized herein revealed a different gene response in rat organs depending on the organ considered. Interestingly, liver, kidneys and testes showed a different level of constitutive MTs: liver and testes held a high amount of MT transcripts, much greater than that determined in kidneys. The high constitutive MT level seems to be strictly related to the pesticide-induced gene response: when the MT gene expression is basically high, the corresponding genes were downregulated; conversely, when transcripts were poorly represented, the MT genes were upregulated. In light of these results, it can be assumed that in rat liver and testes, MT genes play a pivotal role in physiological processes; following an oxidative stress injury, the protective machinery of cells shifts toward the expression of more powerful antioxidant enzymes or proteins with a central role in cellular metabolism. Conversely, in kidneys, the cytoprotective antioxidant machinery involves the ROS scavenger function of MTs, leaving the MT genes responsive to oxidative stress injury. Considering that MTs are often used as a biomarker of cellular damage following environmental pollution [24,27,60,61], these findings also underlined the importance of focusing on the appropriate tissues during biomonitoring studies and knowing the effective content of constitutive MTs in the analyzed tissues to avoid collecting misleading reports.

The drop in MT levels recorded in the liver and testes could also be due to a decrease in free zinc and copper ions, which would no longer be able to induce the synthesis of MTs via the metal-activated transcription factors. The imbalance of reactive oxygen species and the cellular oxidative stress produced by both fatty acids and lipophilic pesticide DDE induce a cascade of events beginning with the peroxidation of membrane lipids, leading to a sharp increase in the activity of metalloenzymes. Trace metal elements, in particular zinc, could be sequestered by these metalloenzymes, such as SOD. It must also be stressed that there might be a different availability of a metal, starting from its entry into cells. In fact, a negative correlation has been demonstrated in the serum levels of trace metals and lipid metabolism products (cholesterol, triglycerides, lipoproteins) [62].

However, the collected data suggested that in the liver and kidneys of the animals from DDE, HF and HF + DDE groups, MT proteins maintained a cytoprotective function, in particular protecting the DNA. Indeed, in the rats fed a high-fat diet and in the DDE-administered rats, a marked increase in the MT content was observed in the nuclei of both hepatic and renal cells, with a concomitant drop in the cytoplasmic protein level. The presence of MTs in nuclei occurring through a nuclear translocation of proteins is considered a cellular mechanism engaged to protect DNA from hydroxyl radical attacks [22,57]. Thanks to the ability of these proteins to donate zinc to several enzymes, a high level of nuclear MTs has also been related to the increased zinc requirement for metalloenzymes and transcription factors during fast cellular metabolism and growth [63,64,65,66].

Taken together, the data show a different involvement of MTs in protecting tissues from HF-induced and DDE-induced oxidative stress, suggesting that different types of cells use different strategies against pro-oxidant species (Table 1). MTs can be abundant in the cells and decrease following a non-metal-induced stress (liver and testes), or increase under oxidative stress (kidneys); finally, they can be mainly cytoplasmic, but can also move to the nucleus.

The data also show that there is no additive or synergistic effect between DDE contamination and an HF diet; on the contrary, high intake of saturated fatty acids ameliorates the DDE-induced damage when administered together. This result allows us to hypothesize that the lipophilic DDE is partly sequestered in the fat deposits that occur in tissues of the rats fed a high-fat diet, thus decreasing the cellular availability of the pesticide. Further studies are needed to confirm our hypothesis and verify the appearance of more damage following the release of DDE from fat deposits that could be induced by a period of caloric restriction. At the same time, further studies are needed to shed more light on the role played by metals, in particular on the connections between the metabolism of lipophilic substances (toxic or not) and lipids on the one hand and the uptake and homeostasis of metallic micronutrients on the other.

## Figures and Tables

**Table 1:** Metallothionein gene expression and protein levels in tissues of rats treated with p,p-dichlorodiphenyldichloroethylene (*p*,*p*-DDE) or p,p-dichlorodiphenyltrichloroethane (*p*,*p*-DDT), alone or in combination with a high-fat diet (HFD).

Metallothionein Gene Expression		HFD	HFD + *p*,*p*-DDE	*p*,*p*-DDE	*p*,*p*-DDT
**Migliaccio et al., 2019.** [56]	LIVER	↓	NTD ↓	NTD ↓	
	TESTES	↓	NTD ↓	NTD ↓	
	KIDNEYS	↓	NTD ↑	NTD ↑	
**Marouani et al., 2017.** [45]	TESTES				HD ↓
**Metallothionein Protein Levels**		**HFD**	**HFD + *p*,*p*-DDE**	***p*,*p*-DDE**	***p*,*p*-DDT**
**Migliaccio et al., 2019.** [56]	LIVER Total homogenate Cytosolic fraction Nuclear fraction	↓ ↓ ↑	NTD ↓ ↓ ↑	NTD ↓ ↓ ↑	
**Migliaccio et al., 2019.** [57]	TESTES Total homogenate	↓	NTD ↓	NTD ↓	
**Migliaccio et al., 2019.** [56]	KIDNEYS Total homogenate Cytosolic fraction Nuclear fraction	↓ - ↑	NTD - - ↑	NTD - - ↑	
**Marouani et al., 2017.** [46]	KIDNEYS Total homogenate	-			HD ↓
**Ben Miled et al., 2017.** [49]	LIVER Total homogenate	-			HD ↑

NTD: non-toxic dose (10 mg/kg bw, administrated orally); HD: high doses (50–100 mg/kg bw, intraperitoneal injection).
